# Integrated Transcriptomic, Proteomic, and Metabolomic Analysis of a Chromosome Segment Substitution Line Reveals the Regulatory Mechanism Governing Fatty Acids and Storage Proteins in Soybean Seeds

**DOI:** 10.3390/genes17040432

**Published:** 2026-04-08

**Authors:** Huidong Qi, Xue Han, Jingyi Huang, Xiaoxia Wu, Jianchun Han

**Affiliations:** National Research Center of Soybean Engineering and Technology, Northeast Agricultural University, Harbin 150030, China; qihuidong2026@126.com (H.Q.); hanxue1860@126.com (X.H.); ohh2026@126.com (J.H.)

**Keywords:** soybean seed, storage protein, fatty acid, CSSLs, multi-omics analysis

## Abstract

**Background/Objectives**: The significant negative correlation between protein and oil content in soybean seeds is a long-standing bottleneck for conventional breeding. Its root cause lies in insufficient understanding of related molecular regulatory processes. **Methods**: We selected the CSSL_R19, a chromosome segment substitution line, to thoroughly investigate the intrinsic effects of the substituted segment on the high seed storage protein (SSP) and low fatty acid (FA) phenotype. Transcriptomic, proteomic, and metabolomic analyses were performed on the recurrent parent and R19. **Results**: A total of 1821 differentially expressed genes (DEGs), 12 differentially expressed proteins (DEPs), and 10 differentially accumulated metabolites (DEMs) were detected. Subsequently, an integrative examination of the data demonstrated that 28 DEGs, 5 DEPs, and 4 DEMs participated in biological processes such as carbohydrate metabolism, lipid degradation, as well as protein synthesis and transport. Mechanistically, down-regulation of PGM reduces the carbon source supply for FA synthesis; up-regulation of LOX, LACS, ACX, and KAT promotes FA degradation. SRP, SAR1, and HSP70 are involved in the synthesis and transport of SSP. Crucially, qRT-PCR validation performed on all 28 core DEGs showed that their expression trends were highly consistent with the transcriptome data, confirming the reliability of the findings. **Conclusions**: In conclusion, we propose a potential regulatory network that enhances SSP accumulation and reduces FA content. Altogether, these findings advance our understanding of storage compound accumulation in soybeans and guide future breeding strategies.

## 1. Introduction

Soybeans (*Glycine max* L. Merr.) are the primary source of vegetable oil combined with protein worldwide. Within soybean seeds, the protein fraction often expands between 35 and 40, which corresponds to the fraction of protein found in everyday cereals. Soybean protein has a relatively balanced composition, containing all 20 amino acids required by the human body. With the exception of methionine, which has a slightly lower content, the remaining amino acids are relatively abundant. As a result, soybean protein can largely substitute proteins found in meat, eggs, and dairy products, making it an ideal source of plant-based protein [1]. Soybean oil, which is obtained from seeds with an oil content of about 18% to 22%, is one of the most widely produced and used edible oils in the world [2]. The soybean seed storage protein and oil content constitute important sources of human nourishment and the development of livestock feeding. With increasing demands from consumers and production needs, the development of breakthrough cultivars that are both high in protein and oil content has been a fundamental goal in soybean genetic improvement.

SSPs and FAs are the major storage compounds in soybean seeds. Understanding networks of gene regulation that control accumulation of storage compounds is a key preliminary step to achieving successful molecular breeding in soybeans. SSPs constitute the majority of proteins in soybean seeds; the main forms include 11S globulin glycinin and 7S globulin b-conglycinin, which account for about 65–80% of the total stored protein in seeds [3]. Over the past several decades, researchers have made significant advances in identifying the molecular and gene organizational structure as well as the regulatory mechanisms of soybean storage proteins, gradually clarifying the role they play in seed development, germination, and the formation of seed quality [4]. With the advancement of proteomics and functional genomics, it has been found that changes in the abundance or subunit composition of β-conglycinin and glycinin can affect the overall attributes of grain protein quality [5,6]. In addition, ectopic expression of the *QQS* gene in soybean seeds increases SSP content by 8–10% by reducing starch accumulation [7]. At the same time, genetics and breeding studies have identified key QTLs and regulatory networks governing seed protein content, providing targets for improving soybean nutritional quality while maintaining yield [8,9].

Five major FAs are predominantly found in soybean seeds: stearic acid (SA, C18:0, 3–5%), palmitic acid (PA, C16:0, 10–12%), α-linolenic acid (ALA, C18:3, 7–10%), oleic acid (OA, C18:1, 20–25%) and linoleic acid (LA, C18:2, 50–55%) [10]. The fatty acid composition of soybeans directly affects their nutritional value. Their health benefits and oxidative stability are primarily determined by their unsaturated fatty acid profile. Knockout of *GmPDCTs* increases OA levels at the expense of polyunsaturated fatty acids, leading to enhanced oil stability [11]. *GmFAD2*, a plant homolog of the desaturase 2 gene, is a key regulator of unsaturated FA composition and the procurement of seed oil in soybeans. Suppression of *GmFAD2* results in a significant increase in OA content and a decrease in LA content [12]. Continuous optimization of the FA profile and mitigating the inverse relationship between protein and oil accumulation has paved the way for functional soybean oil with high OA and improved stability. This provides theoretical and technical support for improving the nutritional quality and shelf-life of edible oils.

The synthesis and accumulation of SSPs and FAs are coordinated and regulated by a complex transcriptional network. The transcription factor WRINKLED1 (WRI1) is a central regulator of fatty acid biosynthetic gene expression, directly activating multiple target genes involved in fatty acid synthesis, including those encoding ACCase and FAS components, and forms a positive regulatory circuit with the seed maturation transcription factor LEAFY COTYLEDON1 (LEC1) to promote the accumulation of storage lipids [13]. Meanwhile, master regulators such as LEC1, LEC2, FUS3, and ABI3 (AFL-type factors) are involved in the broad regulation of seed storage protein genes and the seed maturation process [14,15]. Carbohydrates play a pivotal role in allocating carbon resources between these two biosynthetic pathways. Sucrose produced by photosynthesis in leaves is transported via the phloem to developing seeds and metabolized through glycolysis to generate acetyl-CoA and phosphoenolpyruvate (PEP), which provide common carbon skeletons for the biosynthesis of fatty acids and amino acids [16]. Notably, a pronounced negative correlation exists between storage protein and FA contents, suggesting a competitive mechanism for carbon and nitrogen resources between the two pathways [17]. This trade-off presents a major challenge to simultaneously improve both the protein and oil content in soybean breeding. Although considerable progress has been made in understanding these regulatory networks, the key molecular mechanisms governing the coordinated synthesis and deposition of storage proteins and fatty acids remain largely unclear, highlighting the need for further in-depth investigation through integrated multi-omics approaches.

Integrative analysis of transcriptomics, proteomics, and metabolomics enables the systematic dissection of the entire cascade from gene expression to metabolic products, precisely uncovering the complex regulatory networks underlying the formation of plant traits. Multi-omics has now become a core strategy in plant science for deciphering complex biological processes. The integration of proteomics with transcriptomics has, for instance, uncovered the mechanisms of molecular regulation governing oil and protein content in soybean seeds [18]. The assembly of co-expression networks in maize by integrating proteomic and transcriptomic data facilitated identification of regulatory factors controlling synthesis of storage proteins and clarified the rebalancing process of storage proteins [19]. The integration of metabolomics with transcriptomic and genomic datasets has led to the identification of novel QTLs together with candidate genes participating in the control of protein content in soybeans [20]. Combined transcriptomic and metabolomic analyses have also been extensively employed in the dissection of regulatory mechanisms underlying various biological processes in soybeans, including drought stress, shade tolerance, and nutritional quality [21,22,23,24]. Together, these results underscore the fact that multi-omics integration surpasses the limitations of individual omics approaches, offering a comprehensive view of gene and protein interactions within complex biological networks.

Chromosome segment substitution lines (CSSLs), first used in tomatoes in 1994, have proved to be invaluable genetic resources for dissecting the complexity of crop characteristics [25]. In its ideal form, a CSSL has one or a few donor-derived chromosome fragments on a whole recurrent parental background [26]. Although CSSLs have been extensively employed for fine-mapping of genetic loci underlying various agronomic traits (e.g., yield, plant architecture, and quality) in multiple crops [27,28,29,30], their application in soybeans remains limited [31]. A CSSL population (194 lines) derived from wild soybean ZYD00006 (donor) and cultivated soybean SN14 (recipient) was previously constructed in our laboratory [32]. In the present study, based on analyses of seed FA and SSP contents, we screened and identified a CSSL line exhibiting high SSP and low FA content. We then integrated transcriptomic, proteomic, and metabolomic datasets to construct a previously unknown regulatory network governing the synthesis and deposition of FAs and SSPs in seeds. Our findings provide both practical genetic materials for soybean breeding and fundamental insights into the mechanistic understanding of seed nutrient accumulation.

## 2. Materials and Methods

### 2.1. Plant Materials

Field cultivation took place for the population of CSSLs originally assembled by our laboratory at the Xiangyang Farm in Harbin, China (45.75° N, 126.53° E) [32]. The planting patterns and cultivation practices followed those described by Qi et al. [33]. After measuring the SSP and FA contents of the CSSL population in 2020, we ultimately selected CSSL_R19, which exhibited significant differences from the recurrent parent SN14. Three replicates of dry seeds were collected from both R19 and SN14 after harvest for subsequent analyses.

### 2.2. Measurement of Seed FA Profile and SSP Content

The FA profile of soybeans was analyzed in accordance with the GC method alongside the methyl esterification of fatty acids [34] with some minor modifications. After sieving 60-mesh soybean powder (5 mg), it was combined with 100 mg heptadecanoic acid; this combination was then used with the extraction solvent (2.5% H_2_SO_4_/MeOH [*v*/*v*]). Following 1 h incubation at 85 °C, the mixture was centrifuged for 10 min at 5975× *g*. The supernatant was then discarded, and subsequent pellet treatment was performed using 700 μL hexane with 150 μL of 0.9% saline. Drying at ambient temperature allowed the FAMEs to be redissolved in ethyl acetate (400 μL), enabling GC analysis on the Agilent 7890B system.

The soybean SSP content was determined by analyzing 50 mg of filtered soybean dry powder using an NDA702 Dumas analyzer (VELP Scientifica Srl, Usmate, Italy) [35].

### 2.3. RNA Sequencing Analysis

We performed total RNA extraction in samples and subsequent purification using TRIzol reagent (Invitrogen, Carlsbad, CA, USA). Once quality assessment was finished, fragmentation was conducted on 50 μg of purified mRNA. Sequencing was performed via the Illumina NovaSeq 6000 system using NovaSeq Control Software (NCS) v1.7.0 and Real Time Analysis (RTA) v3.4.5, and paired-end reads were produced, measuring 150 bp. Analysis was subsequently conducted on RNA sequencing data, which mapped initial read counts to a reference genome sequence. Read count quantification and FPKM computation were also performed [33]. Identification of DEGs was performed with the threshold |log_2_FC| > 1 (*p* < 0.05, Student’s *t*-test). A soybean reference genome (Wm82.a2.v1) was used along with associated annotation files derived from Phytozome (https://phytozome-next.jgi.doe.gov/info/Gmax_Wm82_a2_v1, accessed on 20 April 2025).

### 2.4. Proteomics Analysis

#### 2.4.1. Protein Extraction and Digestion

Lysis and protein extraction in R19 and SN14 were performed using the lysis buffer SDT (4% SDS, 100 mM DTT, 150 mM Tris-HCl, pH 8.0). Protein concentration was determined using the BCA Protein Assay Kit (Bio-Rad, Hercules, CA, USA), and protein digestion (300 μg per sample) was conducted following the FASP procedure as described by Han et al. [36]. The resulting peptides were labeled with TMT reagents according to the manufacturer’s instructions (Thermo Fisher Scientific, Waltham, MA, USA). The multiplex-labeled samples were fractionated using the Pierce High pH Reversed-Phase Peptide Fractionation Kit (Thermo Fisher Scientific, Waltham, MA, USA), dried, and then subjected to LC-MS analysis.

#### 2.4.2. LC-MS/MS Analysis

LC-MS analysis was performed on a Q Exactive mass spectrometer coupled to an Easy nLC system (Thermo Fisher Scientific, Waltham, MA, USA). Peptides were loaded onto a trap column (100 µm × 20 mm, 5 µm, C18, Dr. Maisch GmbH, Ammerbuch, Germany) and then separated on an analytical column (75 µm × 150 mm, 3 µm, C18, Dr. Maisch GmbH, Ammerbuch, Germany) at a flow rate of 300 nL/min using a linear gradient of buffer A (2% acetonitrile with 0.1% formic acid) and buffer B (90% acetonitrile with 0.1% formic acid) over 90 min. Data-dependent acquisition was performed on the mass spectrometer for 90 min with the following parameters: MS1 scan range of 300–1800 *m*/*z*, resolution of 60,000 at *m*/*z* 200, automatic gain control (AGC) target of 3 × 10^6^, and maximum injection time of 50 ms. The top 20 most abundant precursor ions from each full scan were selected for MS2 analysis, with MS2 resolution set to 45,000 at *m*/*z* 200, an AGC target of 1 × 10^5^, maximum injection time of 50 ms, isolation window of 1.2 *m*/*z*, and normalized collision energy of 32 using higher-energy collisional dissociation (HCD) [37].

#### 2.4.3. Data Processing

Importation of raw data into Thermo Fisher Scientific’s Proteome Discoverer (v2.4) enabled protein recognition in conjunction with quantification. Data downloading was performed from the Uniprot_Glycine database (https://www.uniprot.org/taxonomy/3847, accessed on 18 May 2025). The initial search parameters were as follows: precursor mass tolerance of 10 ppm; enzymatic cleavage rule set to Trypsin/P, with a maximum of two missed cleavage sites allowed; and a fragment mass tolerance of 20 ppm. Fixed modifications were carbamidomethylation of cysteine residues, TMT10plex labeling of lysine residues, and TMT10plex labeling of peptide N-termini. Variable modifications were methionine oxidation and protein N-terminal acetylation. The minimum peptide length was six amino acids, and each protein was required to have at least one unique peptide. The false discovery rate (FDR) for both peptide and protein identification was set to 1%. Quantification was performed using TMT reporter ion intensities.

#### 2.4.4. Data Analysis and Screening of Differentially Expressed Proteins

Bioinformatics analysis was performed using Perseus software (Version 2.1.6.0; https://maxquant.org/perseus/, accessed on 20 May 2025) and R statistical software (Version 4.2.0; R Core Team, 2022; https://www.r-project.org/, accessed on 25 June 2025). Differentially expressed proteins (DEPs) were screened with a threshold of FC > 1.20 or <0.83 (Student’s *t*-test: *p* < 0.05).

### 2.5. Metabolomics Analysis

#### 2.5.1. Sample Preparation and Extraction

Lyophilization of samples preceded their grinding via a mill. Homogenization was conducted on a 100 mg portion of freeze-dried powder within a 1.2 mL extraction solvent (70% methanol). The mixture underwent vortexing (every 30 min for 30 s with six repetitions) followed by placement in a refrigerator at 4 °C for 12–16 h. Centrifugation of the mixture was carried out at 12,000 rpm for 10 min; after that, filtration of the obtained supernatant was performed on a 0.22 mm microporous membrane (SCAA-104).

#### 2.5.2. UPLC-MS/MS Acquisition Conditions

The filtrate was utilized for UPLC-MS/MS analysis (UPLC: SHIMADZU Nexera X2, Shimadzu Corporation, Kyoto, Japan; MS: Applied Biosystems 4500 Q TRAP, AB Sciex, Framingham, MA, USA). QqQ and LIT acquisition modes were carried out on a Q TRAP MS platform (AB4500 Q TRAP UPLC/MS/MS System, Framingham, MA, USA), with control exerted by Analyst 1.6.3 software (AB Sciex, Framingham, MA, USA). The parameters of the electrospray ionization (ESI) source were as follows: source type, turbo spray; source temperature, 550 °C; ion spray voltage, 5500 V in positive ion mode and –4500 V in negative ion mode; ion source gas I, gas II, and curtain gas pressures set to 50, 60, and 25.0 psi, respectively; and collision-activated dissociation (CAD) set to high. Instrument tuning and mass calibration were performed using 10 μmol/L and 100 μmol/L polypropylene glycol solutions in QQQ and LIT modes, respectively. QQQ scans were acquired in multiple reaction monitoring (MRM) mode with medium collision gas (nitrogen). The declustering potential (DP) and collision energy (CE) for each MRM transition were further optimized. Corresponding MRM transitions were set for monitoring according to the metabolites eluted in each time period.

#### 2.5.3. Metabolite Identification and Differential Screening

Metabolite annotation was based on an in-house database (MWDB, Wuhan Metware Biotechnology Co., Ltd., Wuhan, China). Through an intelligent secondary spectral matching method, the secondary mass spectra and retention times of metabolites in the samples were compared with those in the database. Metabolite identification was based on accurate mass, MS^2^ fragments, isotopic distribution, and retention time, with MS^2^ mass tolerance set to 2 ppm and 5 ppm, respectively. Metabolites without authentic standards were identified by comparison with MS^2^ spectra from public databases or the literature, and some metabolites lacking standard secondary spectra were identified based on empirical inference.

Differentially expressed metabolites (DEMs) were screened with thresholds of VIP ≥ 1 and |FC| > 2. VIP values were extracted from the OPLS-DA results, which were generated using the MetaboAnalystR package in R software.

### 2.6. Bioinformatics Analysis

Databases that were used in annotation included GO (http://geneontology.org/, accessed on 3 June 2025), along with KEGG (https://www.kegg.jp/, accessed on 3 June 2025) and the UniProtKB/Swiss-Prot (https://www.uniprot.org/, accessed on 3 June 2025). Enrichment analysis was performed for GO functions and KEGG pathways using the FDR-corrected Fisher’s exact test.

### 2.7. Quantitative Real-Time PCR Analysis

Soybean seeds were ground into fine powder in liquid nitrogen using a pre-chilled mortar and pestle. Total RNA was extracted using the TRIzol Reagent (Invitrogen, Carlsbad, CA, USA) following the manufacturer’s protocol. RNA concentration and purity were determined spectrophotometrically (NanoDrop 2000, Thermo Fisher Scientific, Waltham, MA, USA), and RNA integrity was verified by 1% agarose gel electrophoresis. In total, 1 μg of total RNA was reverse-transcribed using the HiScript II qRT SuperMix (+gDNA wiper) kit (Vazyme, Nanjing, China). qRT-PCR was performed on a Light Cycler 480 System (Roche Diagnostics, Basel, Switzerland) using the 2 × ChamQ Universal SYBR qPCR Master Mix (Vazyme, Nanjing, China). Three technical replicates were analyzed for each sample, and *GmActin4* was used as the internal reference gene. The relative expression levels of target genes were calculated using the 2^−∆∆CT^ method [38]. Results are presented as the mean of three biological replicates. The primer sequences are listed in Table S1. Statistical significance was assessed using Student’s *t*-test in Microsoft Excel, with *p* < 0.05 considered statistically significant.

### 2.8. Statistical Analysis and Graphical Display

The statistical analysis of phenotypic data was performed using SPSS software (version 17.0; IBM Corp., Armonk, NY, USA) with a two-tailed independent Student’s *t*-test. Prior to analysis, the data were assessed for normality (Shapiro–Wilk test) and homogeneity of variances (Levene’s test); both assumptions were satisfied. A *p*-value < 0.05 was considered statistically significant. Results are presented as the mean ± standard deviation (SD). Histograms were generated using Microsoft Excel (2019). Heatmaps, volcano plots, and bubble plots were created using the pheatmap and ggplot2 packages in R software (Version 4.2.0; R Core Team, 2022; https://www.r-project.org/, accessed on 25 June 2025).

## 3. Results

### 3.1. Selection of a CSSL According to SSP and FA Content

**Figure 1:**
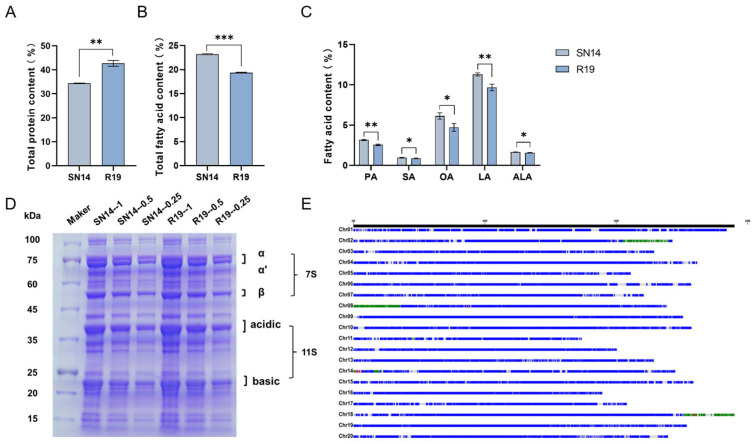
Genotypes paired with phenotypes for comparison between SN14 and R19. (**A**) Total protein content subjected to comparative evaluation. (**B**) Assessment performed comparatively on total fatty acid amounts. (**C**) Fatty acid profiles examined in a comparative fashion. (**D**) Protein profiles investigated comparatively through SDS-PAGE for serial dilutions (1×, 0.5×, and 0.25×). (**E**) Genomic overview of the substitution region of ZYD00006 in R19. Blue segments trace back to the recurrent parent genome of SN14; green and red segments correspond to the donor parent genome of ZYD00006; and gray indicates genomic gaps. Statistical significance is indicated by asterisks (* *p* ≤ 0.05; ** *p* ≤ 0.01; *** *p* ≤ 0.001).

After quantifying SSP and FA in the CSSL population, we selected R19, which differed significantly from the recurrent parent SN14. The total protein content of R19 was 8.37% higher than that of SN14 (Figure 1A). Quantitative analysis of seed proteins by the gradient SDS-PAGE revealed that R19 had greater amounts of proteins corresponding to the 7S and 11S levels (Figure 1D). In contrast, R19 exhibited a 3.83% reduction in the total FA content compared to SN14 (Figure 1B). The five measured fatty acids were all reduced relative to SN14. Among them, the maximum difference in linoleic acid content was 1.62%, and the minimum difference in stearic acid and linolenic acid content was 0.08% (Figure 1C). Resequencing results showed that R19 contained five homozygous introgression fragments from ZYD00006 (Figure 1E), which were located on chromosomes 2, 8, 11, 14, and 18, respectively. Based on the above results, R19 and SN14 were selected for multi-omics analysis.

### 3.2. RNA-Seq Profiling of R19 and SN14

RNA-seq sequencing conducted on R19 with SN14 produced nearly 300 M of clean data overall. Following quality control, mapping rates ranged between 94.56% and 95.38% (Table S2). A global-scale examination determined the expression of 35,178 genes (Table S3). Among the 1821 DEGs detected, 1578 exhibited up-regulation while 243 showed down-regulation in R19 (Table S4, Figure S1A). Regions of chromosomal substitution associated with R19 contained 42 DEGs (Table S5). Annotation of DEGs was performed, and 39 Gene Ontology (GO) terms were identified that met FDR < 0.05, which featured 18 biological process (BP) terms, 10 cellular component (CC) terms, and 11 molecular function (MF) terms (Figure S1B). Terms linked with seed storage substances included glycolytic process (GO:0006096), lipid metabolic (GO:0006629), carbohydrate metabolic process (GO:0005975), amino acid binding (GO:0016597), amino acid metabolic (GO:0006520), and fatty acid biosynthetic (GO:0006633) (Figure S1C). DEGs underwent KEGG annotation covering glycolysis (ko00010), fatty acid biosynthesis (ko00061), fatty acid metabolism (ko01212), fructose and mannose metabolism (ko00051), starch and sucrose metabolism (ko00500), tyrosine metabolism (ko00350), arginine and proline metabolism (ko00330), linolenic acid metabolism (ko00592), phenylalanine, tyrosine and tryptophan biosynthesis (ko00400), lysine biosynthesis (ko00300), arginine biosynthesis (ko00220), biosynthesis of amino acids (ko01230), fatty acid degradation (ko00071) and the citrate cycle (ko00020) (Figure S1D).

### 3.3. Proteomics Analysis of R19 and SN14

Proteomic examination of SN14 and R19 identified 3415 proteins paired with 18,055 peptides (Tables S6 and S7). Significant difference analysis permitted detection of 12 DEPs in total (Table S8). Among them, 4 DEPs showed significant up-regulation, whereas 8 DEPs exhibited significant down-regulation (Figure S2A). Grouping occurred for these DEPs depending on accumulation patterns (Figure S2B). Annotation employing GO was performed on the DEPs. The annotated functions were categorized into 44 BP terms, 29 MF terms and 7 CC terms (Figure S2C), including protein transport (GO:0015031), peptide transport (GO:0015833), the carbohydrate metabolic process (GO:0005975), nitrogen compound transport (GO:0071705), organic substance metabolic process (GO:0071704) and protein binding (GO:0005515). Application of KEGG annotation also identified enriched pathways linked to seed storage, including starch and sucrose metabolism (ko00500) together with linoleic acid metabolism (ko00591) and galactose metabolism (ko00052) (Figure S2D).

**Figure 2:**
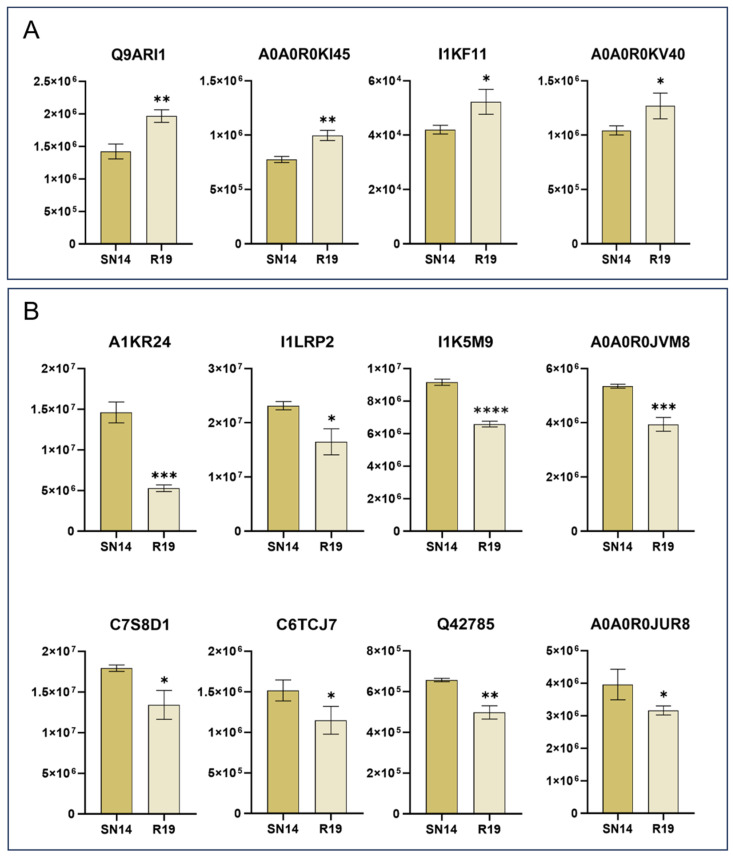
Comparative analysis of DEP abundances between R19 and SN14. (**A**) The 4 up-regulated differentially expressed proteins. (**B**) The 8 down-regulated differentially expressed proteins. Statistical significance is indicated by asterisks (* *p* ≤ 0.05; ** *p* ≤ 0.01; *** *p* ≤ 0.001; **** *p* ≤ 0.0001).

The four significantly increased DEPs were Q9ARI1, A0A0R0KI45, I1KF11 and A0A0R0KV40 (Figure 2A). Compared with SN14, the protein that increased the most in R19 was Q9ARI1 (*Glyma.08G102900*) at 38% higher levels. Another notable protein was A0A0R0KV40 (*Glyma.19G263300*), which showed a 21% increase in accumulation. Both proteins are lipoxygenases and are involved in the linoleic acid metabolic pathway (ko00591). The uncharacterized protein A0A0R0KI45 showed the second-highest increase in abundance, with levels 28% higher than those in SN14. I1KF11 is a dihydroorotase with 24% higher accumulation and participates in the pyrimidine metabolic pathway (ko00240). A total of eight DEPs were significantly down-regulated. These were A1KR24 (dehydrin), I1LRP2 (dirigent protein), I1K5M9 (phosphoglucomutase), A0A0R0JVM8 (mannan endo-1,4-beta-mannosidase), C7S8D1 (germin-like protein), C6TCJ7 (uncharacterized protein), Q42785 (nonsymbiotic hemoglobin), and A0A0R0JUR8 (AB hydrolase-1 domain-containing protein) (Figure 2B). Among these, I1K5M9 is encoded by the differentially expressed gene *Glyma.05G237000* and participates in the glycolytic pathway (ko00010). The markedly down-regulated protein C6TCJ7 is involved in the protein export pathway (ko03060). These DEPs likely play key roles in modulating the differential accumulation of seed storage substances between R19 and SN14.

### 3.4. Metabolomics Analyses of R19 and SN14

**Figure 3:**
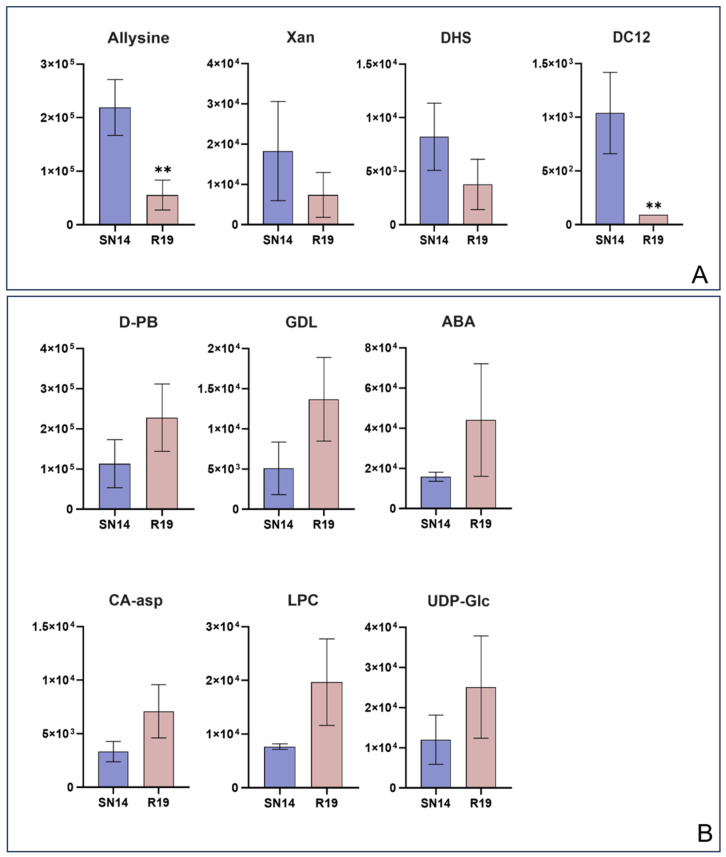
Comparative analysis of DEM abundances between R19 and SN14. (**A**) Four down-regulated differentially expressed metabolites. (**B**) Six up-regulated differentially expressed metabolites. Abbreviations: Allysine (6-Oxo DL-Norleucine), Xan (Xanthine), DHS (3-Dehydroshikimic acid), DC12 (Dodecanedioic acid), D-PB (D-Proline betaine), GDL (D-Glucono-1,5-lactone), ABA (Abscisic acid), CA-asp (N-Carbamoyl-L-aspartate), LPC (LysoPC 20:0), and UDP-Glc (Uridine 5′-diphospho-D-glucose). Statistical significance is indicated by asterisks (** *p* ≤ 0.01).

Metabolomic analysis was conducted on R19 and SN14. We identified 574 metabolites through UPLC-MS/MS analysis coupled with a self-built database (Table S9). Altogether, 10 DEMs were determined; of these, six were significantly up-regulated, and four were significantly down-regulated (Figure 3). These DEMs included two lipids, two organic acids, one saccharide and alcohol, two nucleotides and derivatives, and three amino acids and derivatives (Table S10).

The DEMs were analyzed for KEGG pathway enrichment (Figure S3). Allysine is an amino acid derivative involved in both lysine biosynthesis (ko00300) and degradation (ko00310); its expression trend was down-regulated in both pathways. DHS is an organic acid involved in the phenylalanine, tyrosine, and tryptophan biosynthesis pathway (ko00400), and its expression trend was shown to be down-regulated. UDP-Glc is a critically important nucleotide sugar. By activating glucose, it directly links energy metabolism with storage. Diverse pathways show evidence for the participation of UDP-Glc, encompassing zeatin biosynthesis (ko00908), glycerolipid metabolism (ko00561), and the interconversion of pentose and glucuronate (ko00040). The expression trend of this metabolite is up-regulated in these pathways. CA-asp is a key intermediate in pyrimidine nucleotide biosynthesis. It showed up-regulation and involvement in the pyrimidine metabolism pathway (ko00240). GDL is an intermediate in the pentose phosphate pathway (ko00030), and its expression trend is up-regulated in R19. Xanthine is involved in the purine metabolism pathway (ko00230) and exhibits a down-regulated expression trend. ABA participates in the plant hormone signal transduction pathway (ko04075) and exhibits an up-regulated expression trend. Finally, Dodecanedioic acid is a free fatty acid, and its expression is down-regulated. Compared with SN14, the content of LPC) in R19 was 156% higher.

### 3.5. Integrated Multi-Omics Analysis of R19 and SN14

#### 3.5.1. Integrative Multi-Omics Analysis Uncovers the Regulatory Network Underlying Carbohydrate Metabolism

**Figure 4:**
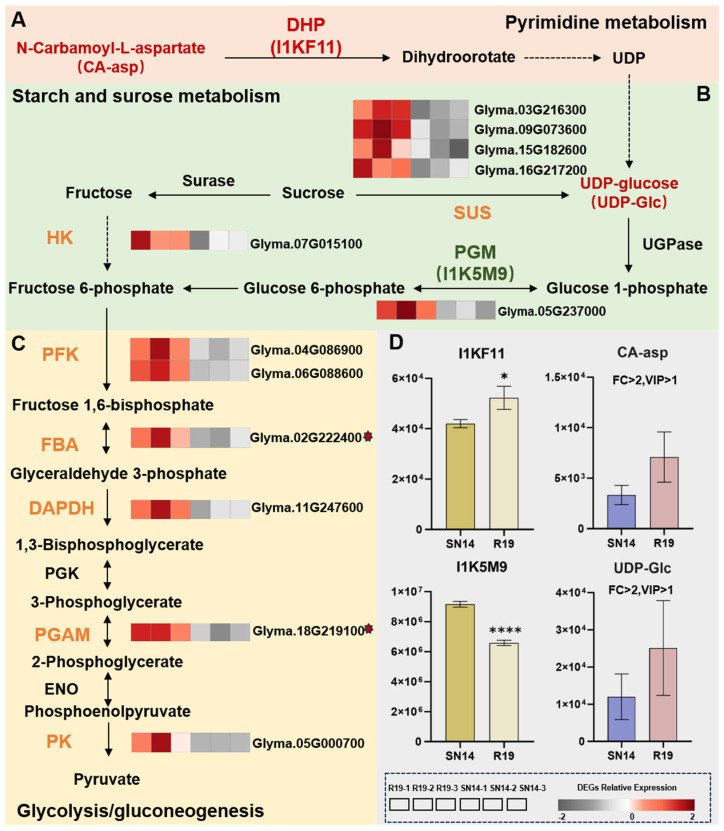
Regulatory pathways involved in carbohydrate metabolism by DEGs, DEPs, and DEMs. (**A**) The pyrimidine biosynthesis pathway. DEP involved DHP (dihydroorotase); DEM involved CA-asp (N-Carbamoyl-L-aspartate). (**B**) The sucrose metabolism pathway. DEP involved PGM (phosphoglucomutase); DEM involved UDP-Glc (Uridine 5′-diphospho-D-glucose); proteins encoded by DEGs: SUS (sucrose synthase) and HK (hexokinase). (**C**) The glycolysis/gluconeogenesis pathway. Proteins encoded by DEGs: PFK (ATP-dependent 6-phosphofructokinase), FBA (fructose-bisphosphate aldolase), DAPDH (Glyceralde-hyde-3-phosphate dehydrogenase), PGAM (phosphoglycerate mutase), and PK (pyruvate kinase). (**D**) Comparative analysis of DEG and DEM abundances. Solid arrows represent direct regulation, dashed arrows represent multi-step processes, and double-headed arrows represent reversible reactions. The heatmap displays the relative expression levels of DEGs, with red indicating upregulation and gray indicating downregulation. Red text indicates up-regulation, and green text indicates down-regulation. Red asterisks denote the DEGs located in the introgressed region of R19. Statistical significance is indicated by asterisks (* *p* ≤ 0.05; **** *p* ≤ 0.0001).

Up-regulated changes were observed in the de novo pyrimidine biosynthesis pathway (ko00240), including the metabolite CA-asp and the protein I1KF11 (Dihydroorotase, DHP). CA-asp is catalyzed by dihydroorotase to produce dihydroorotate, which is ultimately converted into UDP through the de novo pyrimidine nucleotide biosynthesis pathway—a critical precursor for UDP-Glc production [39] (Figure 4A).

In the sucrose metabolism pathway (ko00500), sucrose synthase (SUS), encoded by four up-regulated DEGs, catalyzes the cleavage of sucrose to produce fructose and UDP-Glc. UDP-Glc is catalyzed to produce glucose-1-phosphate [40], and fructose is phosphorylated to fructose 6-phosphate by HK). The resulting phosphatehexoses from both pathways can enter glycolysis [41]. In R19, the abundance of the metabolite UDP-Glc was higher than in SN14. We hypothesize that this increase was due to the significantly up-regulated expression of the differentially expressed gene *Glyma.07G015100*, which encodes HK, while no differential expression was observed for the gene encoding UGPase. The protein I1K5M9, encoded by *Glyma.05G237000*, is a PGM. Its abundance was significantly lower in R19 than in SN14. Glucose 6-phosphate (G6P) is a major precursor for both fatty acid and starch synthesis. PGM is a key regulator in maintaining G6P content. The observed down-regulation of PGM in R19, compared to SN14, suggests a potential indirect contribution to the decrease in FA content (Figure 4B).

Five up-regulated DEGs were uncovered in the glycolysis pathway, encoding PGAM along with GAPDH, FBA, and PFK. Among them, introgression regions harbor *Glyma.18G219100* together with *Glyma.02G222400*. Encoding of PK occurs via *Glyma.05G000700*; this enzyme catalyzes the transfer of phosphate groups from PEP to ADP, thereby producing ATP paired with pyruvate. Irreversible reactions of glycolysis are regulated by PFK, combined with PK and HK. The entry of glucose in glycolysis is governed by HK, and the conclusion of glycolysis is catalyzed by PK. PFK serves as the key rate-limiting enzyme [42] (Figure 4C). Pyruvate originating in glycolysis is conveyed into the plastid and undergoes immediate conversion into Acetyl-CoA mediated by pyruvate dehydrogenase, thereby supplying a carbon backbone essential for FA biosynthesis. Concurrently, pyruvate contributes carbon skeletons to amino acid biosynthesis, either directly or through the TCA cycle [43].

#### 3.5.2. Integrative Multi-Omics Analysis Uncovers the Regulatory Network Underlying Lipid Metabolism

**Figure 5:**
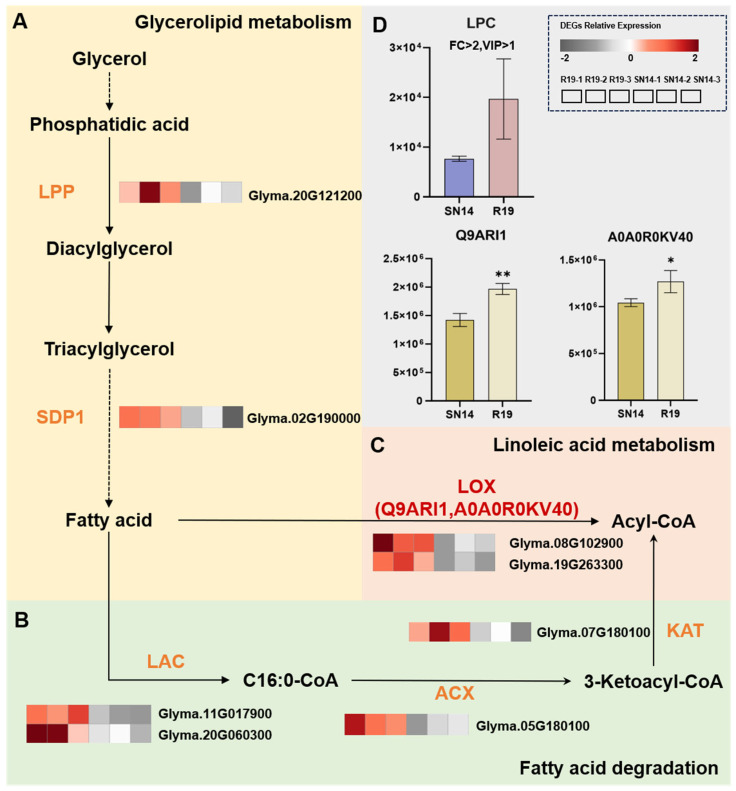
Regulatory pathways involved in lipid metabolism by DEGs, DEPs, and DEMs. (**A**) The glycerolipid metabolism pathway. Proteins encoded by DEGs: LPP (lipid phosphate phosphatase) and SDP1 (sugar-dependent 1). (**B**) The fatty acid degradation pathway. Proteins encoded by DEGs: LAC (long-chain acyl-CoA synthetase), ACX (CoA oxidase), and KAT (peroxisomal 3-ketoacyl-CoA thiolase). (**C**) The linoleic acid metabolism pathway. DEP involved LOX (lipoxygenase). (**D**) Comparative analysis of DEG and DEM abundances. Solid arrows represent direct regulation, dashed arrows represent multi-step processes. The heatmap displays the relative expression levels of DEGs, with red indicating upregulation and gray indicating downregulation. Red text indicates up-regulation. Statistical significance is indicated by asterisks (* *p* ≤ 0.05; ** *p* ≤ 0.01).

Triacylglycerol (TAG) is a major storage lipid in seeds, localized within oil bodies. In the glycerolipid metabolism pathway (ko00561), the expression of DEG (*Glyma.20G121200*), which encodes a lipid phosphate phosphatase (LPP), is significantly up-regulated in R19. This enzyme dephosphorylates phosphatidic acid (PA) to form diacylglycerol (DAG) [44]. In R19, the abundance of metabolite LPC was 2.56 times higher than that in SN14. Studies have shown that the exchange of the base group in phosphatidylcholine (PC) is accomplished through the enzymatic action of phospholipases, which generate LPC by releasing free fatty acids. LPC is converted into PC through the enzymatic function of lysophosphatidylcholine acyltransferase (LPCAT), utilizing acyl-CoA to donate the acyl group to proteins [45]. In the TAG biosynthesis pathway of some oilseeds, DAG can be rapidly converted to PC and vice versa. Phosphatidylcholine:DAG cholinephosphotransferase (PDCT) can efficiently promote this reaction [46]. Esterification of DAG at the *sn-3* site forms the storage lipid TAG (Figure 5A).

The differentially expressed gene *Glyma.02G190000* is up-regulated in R19 and SDP1 lipase, which hydrolyzes TAG to release free FAs and DAG [47]. Four up-regulated DEGs, *Glyma.11G017900*, *Glyma.20G060300*, *Glyma.05G180100* and *Glyma.07G180100*, encode LACS4, LACS8, ACX, and KAT, respectively. These enzymes act as central players in the peroxisomal oxidation of FAs for the biosynthesis of acetyl-CoA. Thus, the increased expression of these genes may promote FA oxidation, thereby reducing the FA content in R19 (Figure 5B). In the linoleic acid metabolism pathway (ko00591), two LOX proteins, Q9ARI1 and A0A0R0KV40, were identified with fold changes in abundance of 1.38 and 1.22, respectively. Additionally, two up-regulated DEGs encoding LOX, *Glyma.08G102900* and *Glyma.19G263300*, were also discovered [48] (Figure 5C).

#### 3.5.3. Integrative Multi-Omics Analysis Uncovers the Regulatory Network Underlying Amino Acid and Storage Protein Synthesis

**Figure 6:**
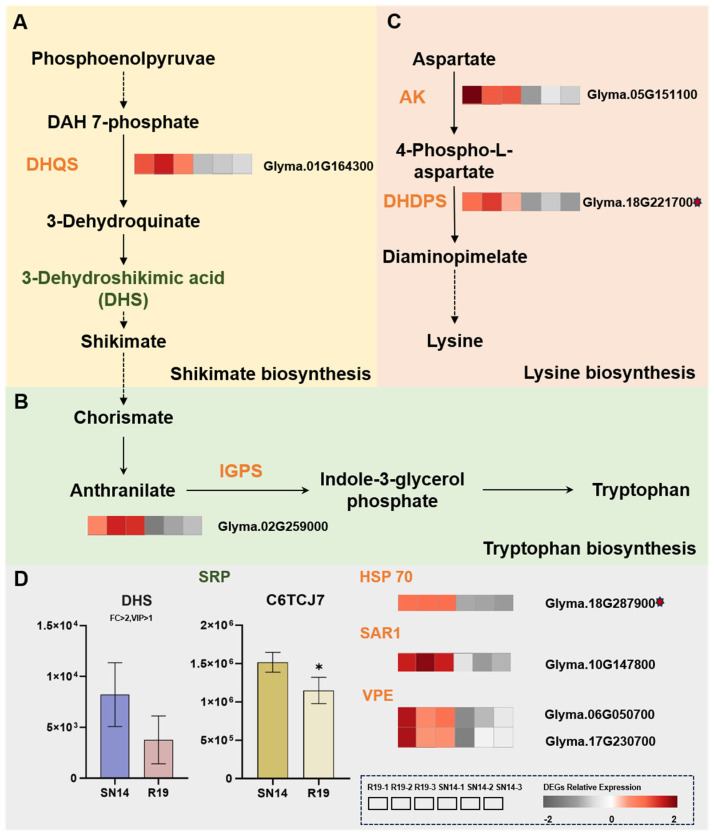
Regulatory pathways involved in amino acid and storage protein synthesis with DEGs, DEPs, and DEMs. (**A**) The shikimate pathway. DEM involved DHS (3-Dehydroshikimic acid); proteins encoded by DEGs: DHQS (3-dehydroquinate synthase). (**B**) The tryptophan biosynthesis pathway. Proteins encoded by DEGs: IGPS (indole-3-glycerol-phosphate synthase). (**C**) The lysine biosynthesis pathway. Proteins encoded by DEGs: AK (aspartate kinase) and DHDPS (dihydrodipicolinate synthase). (**D**) Comparative analysis of DEG and DEM abundances. SRP (signal recognition particle), HSP 70 (heat shock protein 70), SAR1 (secretion-associated Ras-related protein 1), and VPE (vacuolar-processing enzyme) involved in protein synthesis and transport. Solid arrows represent direct regulation, dashed arrows represent multi-step processes. The heatmap displays the relative expression levels of DEGs, with red indicating upregulation and gray indicating downregulation. Green text indicates down-regulation. Red asterisks denote the DEGs located in the introgressed region of R19. Statistical significance is indicated by asterisks (* *p* ≤ 0.05).

In the shikimate pathway, the DEG (*Glyma.01G164300*) was up-regulated. It encodes 3-dehydroquinate synthase, while the down-regulated metabolite DHS serves as an intermediate in the same pathway. Chorismate, the final product of this pathway, serves as the direct precursor for the synthesis of phenylalanine, tryptophan, and tyrosine [49] (Figure 6A). The DEG (*Glyma.02G259000*) in the R19 substituted region encodes indole-3-glycerol-phosphate synthase (IGPS), which is a key enzyme involved in tryptophan synthesis [50]. Up-regulated DEGs promote the biosynthesis of tryptophan, which is a limiting factor for protein synthesis (Figure 6B). Through the investigation of the lysine biosynthesis pathway (ko00300), two up-regulated DEGs were identified: *Glyma.05G151100* and *Glyma.18G221700* (located within the R19 introgression region). These genes encode aspartate kinase (AK) and dihydrodipicolinate synthase (DHDPS), respectively. They are key enzymes in the synthesis of lysine [51,52] (Figure 6C).

The abundance of protein C6TCJ7 (signal recognition particle, SRP) was down-regulated. This protein is responsible for the direct transport of nascent proteins from the ribosome to the endoplasmic reticulum (ER) [53]. Research has revealed that high expressions of SRP selectively restrict the expression of membrane proteins. Therefore, the down-regulated C6TCJ7 protein would promote the reaction [54]. Based on the annotation of the ER protein processing pathway, we identified an up-regulated candidate gene, *Glyma.18G287900*, which encodes HSP70. This gene is located within a substitution segment in R19. Driven by ATP, HSP70 can transiently interact with proteins, thereby preventing misfolding and facilitating the proper folding of some protein molecules through rapid isomerization in the ER [55]. In R19, the up-regulated differentially expressed gene *Glyma.10G147800* encodes SAR1. SAR1 is the core initiator and regulatory switch for COPII-coated vesicle formation. These vesicles are responsible for transporting nascent proteins from the ER to the Golgi network. Subsequently, they are sorted at the trans-Golgi network, enter multivesicular bodies, and finally reach the protein storage vacuoles. After being delivered to the protein storage vacuole, the soybean storage protein precursors undergo further processing to mature and subsequently precipitate or accumulate in a stable manner [56]. Two up-regulated DEGs, *Glyma.06G050700* and *Glyma.17G230700*, encode VPEs. Through the action of VPEs, unprocessed precursor proteins undergo processing to yield their mature forms [57] (Figure 6D).

Collectively, through the integrative analysis of the aforementioned three omics datasets, we elucidated a novel pathway that regulates the fatty acid and storage protein contents in soybean seeds. This pathway contains 28 DEGs, 5 DEPs, and 4 DEMs (Table S11).

### 3.6. Validation of DEGs Involved in Regulatory Networks

**Figure 7:**
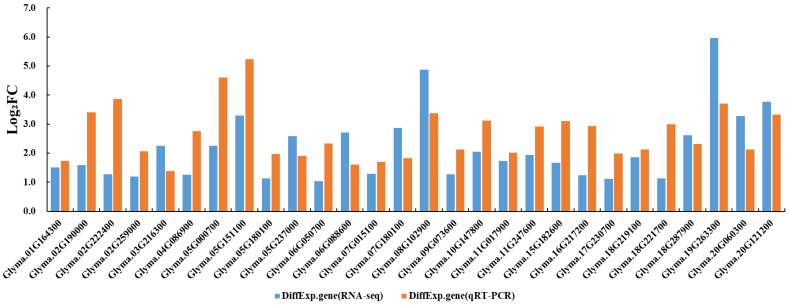
Comparison of DEG expressions between RNA-seq and qRT-PCR. The horizontal axis represents the 28 DEGs involved in the SSP and FA regulatory networks. The vertical axis indicates the magnitude of gene expression changes, where Log_2_FC = Log_2_ (R19 expression/SN14 expression). Blue represents RNA-seq data, and orange represents qRT-PCR data.

To further validate the reliability of the above results, 28 DEGs involved in the SSP and FA regulatory networks were selected for qRT-PCR analysis in this study. The results showed that these genes were all up-regulated in R19, and the expression trends were highly consistent with the transcriptome sequencing data, confirming the reliability of the transcriptome analysis results (Figure 7).

## 4. Discussion

Globally, soybeans supply about 70% of plant-based dietary protein and nearly 30% of vegetable oil, which has direct implications for global food security and livestock feed supply [58]. Soy protein provides all the essential amino acids needed in humans. This high-quality plant protein offers a crucial alternative to animal protein, helping to optimize dietary structures [56]. Soybean oil has a high proportion of unsaturated FAs, with LA and other types constituting the majority of its composition, contributing to reduced blood cholesterol levels and the maintenance of cardiovascular health. Thus, the composition and content of FAs directly determine their nutritional value [59,60]. During seed development, accumulation of protein and oil typically exhibits a negative correlation. Elucidating the regulatory networks that govern protein and oil synthesis, transport, and deposition at the molecular and metabolic levels enables the use of gene editing and molecular design in breeding to enhance soybean quality with precision, ultimately achieving a synergistic increase in both protein and oil content [5].

A set of CSSL populations forms a library providing full genomic coverage of the donor parent. Its core value is to function as a “permanent genetic resource repository” to provide an efficient platform for the mining and mapping of trait–genotype associations [61]. Each line from the library has a highly similar genetic background to the recurrent parent SN14, except for the introduction of a limited number of chromosomal fragments from the wild soybean parent. Compared to single-omics analyses, the combination of multi-omics datasets (transcriptomics, proteomics, and metabolomics) allows the entire molecular cascade from DNA and RNA to proteins and metabolites to be captured. By relating upstream regulatory events with downstream functional and metabolic outcomes via the construction of multi-scale networks, it is possible to better pinpoint the actual regulatory factors and key enzymes regulating specific metabolic pathways. This approach gives rise to a more causal and mechanistic understanding of biological processes [62,63,64]. The application of CSSL populations in multi-omics research started relatively late but has progressed rapidly, and the combination of the two offers the dual advantages of optimized material design and methodological integration. The characteristic feature of CSSLs, namely a “uniform genetic background plus single-segment substitution”, can effectively reduce background interference in the analysis of complex traits, whereas the integrated analysis of transcriptomics, proteomics, and metabolomics can systematically reveal the regulatory processes mediated by target segments at multiple molecular levels. The integration of these two approaches not only improves the accuracy of candidate genes and key regulatory module identification but also provides a more causal framework for elucidating the mechanisms underlying complex trait formation [65]. In this study, we identified an introgression line, R19, characterized by a high SSP and low FA content. Through integrated transcriptomic, proteomic, and metabolomic analyses, we detected 1821 DEGs (42 of which are located within the substituted segment), 12 DEPs, and 10 DEMs. The relatively low number of DEPs and metabolites can be attributed to the clear genetic background and single-fragment substitution characteristic of CSSLs. This distinct feature allowed us to clearly elucidate how differentially expressed molecules from the wild fragment interact synergistically with existing differential molecules in the cultivated soybean background. Consequently, this interaction systematically reprograms the biosynthesis and deposition network of storage proteins and fatty acids, ultimately driving the formation of target traits such as high protein and low oil content.

Multi-omics analysis showed that differentially expressed molecules are enriched in pathways related to carbohydrate and lipid metabolism, protein processing, and protein transport. The protein I1K5M9, encoded by the differentially expressed gene *Glyma.05G237000*, is identified as PGM. Its abundance is significantly lower in R19 compared to SN14, which may explain the differences in fatty acid content found in R19 seeds. PGM is a key regulatory factor in maintaining G6P levels. G6P supplies the carbon backbone for the biosynthesis of both FAs and starch. Although direct observation of significant changes in G6P levels was precluded by the filtering criteria applied in differential metabolite screening, the metabolomic data nonetheless provide important indirect evidence. In all three biological replicates of R19, the abundance of glucose-1-phosphate (G1P) was consistently higher than that of SN14, whereas the abundance of fructose-6-phosphate (F6P) was consistently lower. The accumulation of G1P suggests that its conversion to G6P is impaired, while the reduction in F6P levels, given that G6P is further converted to F6P by phosphoglucose isomerase, indirectly reflects a decrease in G6P supply. These metabolic features are consistent with the observed down-regulation of PGM protein abundance, indicating that carbon flux into glycolysis and fatty acid biosynthesis may be restricted in R19, ultimately leading to a reduction in seed fatty acid content. Taken together, the low expression of PGM may limit the availability of G6P, thereby limiting the carbon skeleton supply required for fatty acid synthesis and consequently impairing lipid accumulation in R19. It has been reported that *pgm* mutant seeds in Arabidopsis exhibit a 40% lower oil content than wild-type seeds [66]. The pyruvate generated by PK in the final step of glycolysis is further converted into acetyl-CoA, either in mitochondria or plastids. This process has a direct effect on the supply of energy and carbon flow through FA synthesis and other amino acid biosynthesis. Changes in central carbon metabolism, in turn, regulate the relative proportions of SSP and FAs [67]. In soybeans, interventions at these key nodes of metabolism often result in phenotypes such as “decreased oil with increased protein” or their combination, pointing to the importance of central carbon metabolism in regulating oil vs. protein accumulation [68]. Deciphering the role of PK in determining the balance between oil and protein content will help breeders precisely alter the composition of seeds to develop better soybean varieties with high levels of both macromolecules.

A subset of the identified DEGs encodes proteins that function in lipid metabolism. For example, SDP1 is a key lipase that initiates TAG hydrolysis [69]. Studies have shown that overexpression of SDP1 reduces TAG content in mutants and redirects the released fatty acids to peroxisomes for β-oxidation [70]. LACS is responsible for converting free fatty acids into acyl-CoA, thereby playing a crucial role in the β-oxidation process within glyoxysomes [71]. This oxidation pathway further involves enzymes such as ACX, MFP, and KAT, which promote the flow of carbon skeletons toward carbohydrate biosynthesis [72,73,74]. In addition, LOX proteins function as key mediators in the degradation of storage lipids and FAs. Previous studies on maize have also confirmed that the inactivation of lipoxygenase *ZmLOX3* increases accumulation of free FAs in mutant kernels [75]. The elevated ABA content observed in the R19 line in this study may also be related to LOX activity. Rober et al. reported that the lipoxygenase family participates in regulating ABA biosynthesis and experimentally demonstrated that lipoxygenase inhibitors suppress ABA accumulation in soybean seedlings [76]. The concerted action of these lipid degradation and oxidation pathways may contribute to the lower fatty acid content in R19.

Using an integrated multi-omics approach, we identified a set of DEGs and DEPs implicated in the regulation of SSP accumulation and amino acid biosynthesis. In higher plants, SSPs typically contain an N-terminal signal peptide. After synthesis on cytoplasmic ribosomes, the signal peptide is specifically recognized by SRP (corresponding to protein C6TCJ7), which targets the protein to the endoplasmic reticulum in a co-translational manner. The SRP pathway is essential for proper transport and high-level accumulation of storage proteins, and its efficiency and fidelity directly influence both the yield and quality of storage proteins in seeds [77]. HSP70 (*Glyma.18G287900*) is a molecular chaperone, playing an important role in the correct folding, assembly, quality control and homeostasis maintenance of storage proteins under high-load synthesis conditions [78]. Moderate up-regulation of HSP70 expression helps increase the efficiency of the storage proteins in the occurrence of soluble proteins and accumulation capacity [79]. SAR1 (*Glyma.10G147800*) is a key rate-limiting factor that regulates the proper localization of SSPs, protein body formation and ultimately seed quality. Its functional deficiency often results in floury endosperm (which is an important molecular basis for such kinds of phenotypes) and abnormal processing of storage proteins [80]. The synchronized action of several processes is probably responsible for the marked increase in SSP content in the R19 line.

**Figure 8:**
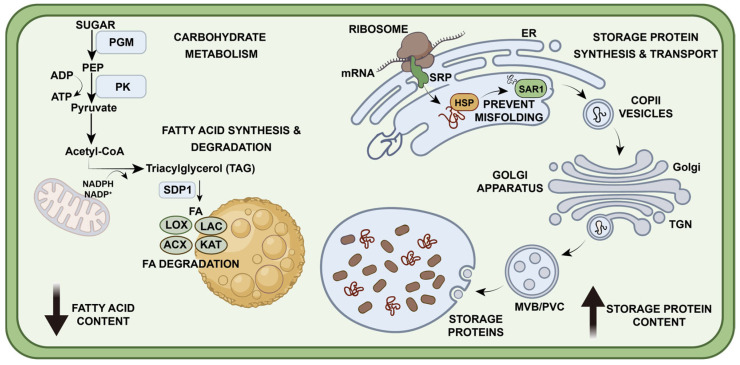
Proposed regulatory network governing SSP and FA accumulation in soybean seeds. Abbreviations: PGM (phosphoglucomutase), PEP (phosphoenolpyruvate), PK (pyruvate kinase), SDP1 (sugar-dependent 1), LOX (lipoxygenase), ACX (Acyl-CoA oxidase), LAC (Laccase), KAT (3-ketoacyl-CoA Thiolase), SRP (signal recognition particle), HSP70 (heat shock protein 70), and SAR1 (secretion-associated Ras-related protein 1).

Previous studies have shown that transcriptomic analysis can elucidate the regulatory networks underlying soybean protein and oil content. LAC has been reported to be associated with lipid metabolism, and HSP is involved in protein folding, which is consistent with our observations. By integrating proteomic and metabolomic analyses with transcriptomic approaches, we identified multiple novel key enzymes and regulatory factors, providing new insights into the mechanisms governing protein and oil accumulation in soybeans [81]. Therefore, we propose a potential regulatory network that can explain the high SSP and low FA content found in R19 (as shown, for example, in Figure 8). The down-regulated PGM limits the flow of carbon into glycolysis, which indirectly limits the primary source of fatty acid synthesis. PK catalyzes the final step of glycolysis and pyruvate generation, which enters the mitochondria and is then converted into acetyl-CoA, which is the direct carbon source of FA biosynthesis. The up-regulated SDP1 catalyzes the hydrolysis of TAG into free FAs and DAGs. Four up-regulated proteins (LOX, LACS, ACX, and KAT) are involved in FA degradation, thereby limiting FA biosynthesis. On the other hand, amino acid skeletons are largely derived from intermediates of glycolysis and the TCA cycle, with certain amino acids (such as alanine, leucine, and valine) directly utilizing pyruvate as a precursor. SRP mediates the translocation of proteins from ribosomes to the endoplasmic reticulum, while HSP70 interacts transiently with proteins to prevent misfolding. Through the activation of SAR1, storage proteins are packaged into COPII vesicles, trafficked to the Golgi network, sorted at the TGN, and ultimately delivered to the vacuole via multivesicular bodies.

While our integrated multi-omics analysis provides a robust foundation for the proposed regulatory model, the current sample size limits the application of quantitative network analysis metrics, such as weighted gene co-expression network analysis (WGCNA). Future studies incorporating developmental time-course data or expanded CSSL populations are essential to construct statistically significant co-expression networks and further validate these regulatory modules quantitatively.

Although the regulatory network requires further validation, our integrated transcriptomic, proteomic, and metabolomic analyses have systematically elucidated the multi-layer regulatory network governing SSP and FA accumulation in soybeans. This study establishes theoretical groundwork and molecular targets for understanding the “high-oil–high-protein” negative correlation and offers scientific support for breeding dual-purpose soybean varieties with high oil and protein.

## 5. Conclusions

This study integrated the transcriptomic, proteomic, and metabolomic analyses of R19 and the recurrent parent. A total of 1821 DEGs, 12 DEPs, and 10 DEMs were identified. Among these, 28 DEGs, 5 DEPs, and 4 DEMs are implicated in diverse biological processes: carbohydrate metabolism, FA oxidation and degradation, and SSP synthesis and transport. Mechanistically, down-regulation of PGM reduces the carbon source supply for FA synthesis. Up-regulation of LOX, LACS, ACX, and KAT promotes the degradation of FAs. SRP, SAR1, and HSP70 are involved in the synthesis and transport of SSP. These findings identify novel molecular mechanisms governing the accumulation of seed storage compounds in soybeans, laying a foundation for the molecular design breeding of high-quality varieties.

## Data Availability

The original contributions presented in this study are included in the article/Supplementary Materials. Further inquiries can be directed to the corresponding authors.

## Supplementary Materials

The following supporting information can be downloaded at https://www.mdpi.com/article/10.3390/genes17040432/s1. Figure S1: Comparative RNA-seq analysis between R19 and SN14. (A) Volcano plot of up-regulated and down-regulated DEGs. (B) GO annotation of DEGs. (C) Bubble plot of GO enrichment of DEGs. (D) Bubble plot of KEGG enrichment of DEGs; Figure S2: Comparative proteomic analysis between R19 and SN14. (A) Volcano plot of up-regulated and down-regulated DEPs. (B) Heatmap clustering of DEPs. (C) Bubble plot illustrating the GO enrichment analysis of DEPs. (D) Bubble plot illustrating the KEGG enrichment analysis of DEPs; Figure S3: The KEGG enrichment analysis of DEMs between R19 and SN14; Table S1: Primer sequences used for quantitative real-time PCR (qRT-PCR) analysis; Table S2: Mapping statistic of RNA-Seq raw data of SN14 and R19; Table S3: All expressed genes annotated from SN14 and R19; Table S4: All differentially expressed genes annotated SN14 and R19; Table S5: DEGs located in the substituted region of R19; Table S6: Identification of peptide by TMT-based quantitative proteomics; Table S7: All expressed proteins annotated between SN14 and R19; Table S8: All differentially expressed proteins between annotated SN14 and R19; Table S9: All expressed metabolites annotated between SN14 and R19; Table S10: All differentially expressed metabolites between annotated SN14 and R19; Table S11: The DEGs, DEPs and DEMs involved the seed storage-related pathway.

## Abbreviations

The following abbreviations are used in this manuscript:

SSP	Seed Storage Protein
FA	Fatty Acid
DEG	Differentially Expressed Gene
DEP	Differentially Expressed Protein
DEM	Differentially Expressed Metabolite
SA	Stearic Acid
PA	Palmitic Acid
ALA	α-Linolenic Acid
OA	Oleic Acid
CSSL	Chromosome Segment Substitution Line
SDS-PAGE	Sodium Dodecyl Sulfate–Polyacrylamide Gel Electrophoresis
BP	Biological Process
CC	Cellular Component
MF	Molecular Function
DHS	3-Dehydroshikimic Acid
UDP-Glc	Uridine 5′-diphospho-D-glucose
CA-asp	N-Carbamoyl-L-aspartate
GDL	D-Glucono-1,5-lactone
ABA	Abscisic Acid
LPC	LysoPC 20:0
D-PB	D-Proline Betaine
Xan	Xanthine
DC12	Dodecanedioic Aicd
DHP	Dihydroorotase
SUS	Sucrose Synthase
HK	Hexokinase
UGPase	UDP-glucose Pyrophosphorylase
PGM	Phosphoglucomutase
PFK	ATP-dependent 6-phosphofructokinase
FBA	Fructose-bisphosphate Aldolase
DAPDH	Glyceraldehyde-3-phosphate Dehydrogenase
PGAM	Phosphoglycerate Mutase
PK	Pyruvate Kinase
G6P	Glucose 6-phosphate
TAG	Triacylglycerol
LPP	lipid Phosphate Phosphatase
PA	Phosphatidic Acid
DAG	Diacylglycerol
LPCAT	Lysophosphatidylcholine Acyltransferase
PDCT	Phosphatidylcholine: DAG Cholinephosphotransferase
SDP1	Sugar-dependent 1
LOX	Lipoxygenase
ACX	CoA Oxidase
LAC	Long chain Acyl-CoA synthetase
KAT	Peroxisomal 3-ketoacyl-CoA Thiolase
IGPS	Indole-3-glycerol-phosphate Synthase
DHDPS	Dihydrodipicolinate Synthase
AK	Aspartate Kinase
DHQS	3-dehydroquinate Synthase
SRP	Signal Recognition Particle
HSP 70	Heat Shock Protein 70
SAR1	Secretion-Associated Ras-related protein 1
VPE	Vacuolar Processing Enzyme
ER	Endoplasmic Reticulum
GC	Gas Chromatography
FAMEs	Fatty Acid Methyl Esters
FPKM	Fragments Per Kilobase of transcript per Million mapped reads
TMT	Tandem Mass Tag
GO	Gene Ontology
KEGG	Kyoto Encyclopedia of Genes and Genomes
FDR	False Discovery Rate
ANOVA	Analysis of Variance
