# Supplementary figures and images for: Integrated Transcriptomic, Proteomic, and Metabolomic Analysis of a Chromosome Segment Substitution Line Reveals the Regulatory Mechanism Governing Fatty Acids and Storage Proteins in Soybean Seeds
Source: Genes (Basel). 2026 Apr 8;17(4):432. doi: 10.3390/genes17040432 (PMC13116479; doi:10.3390/genes17040432)

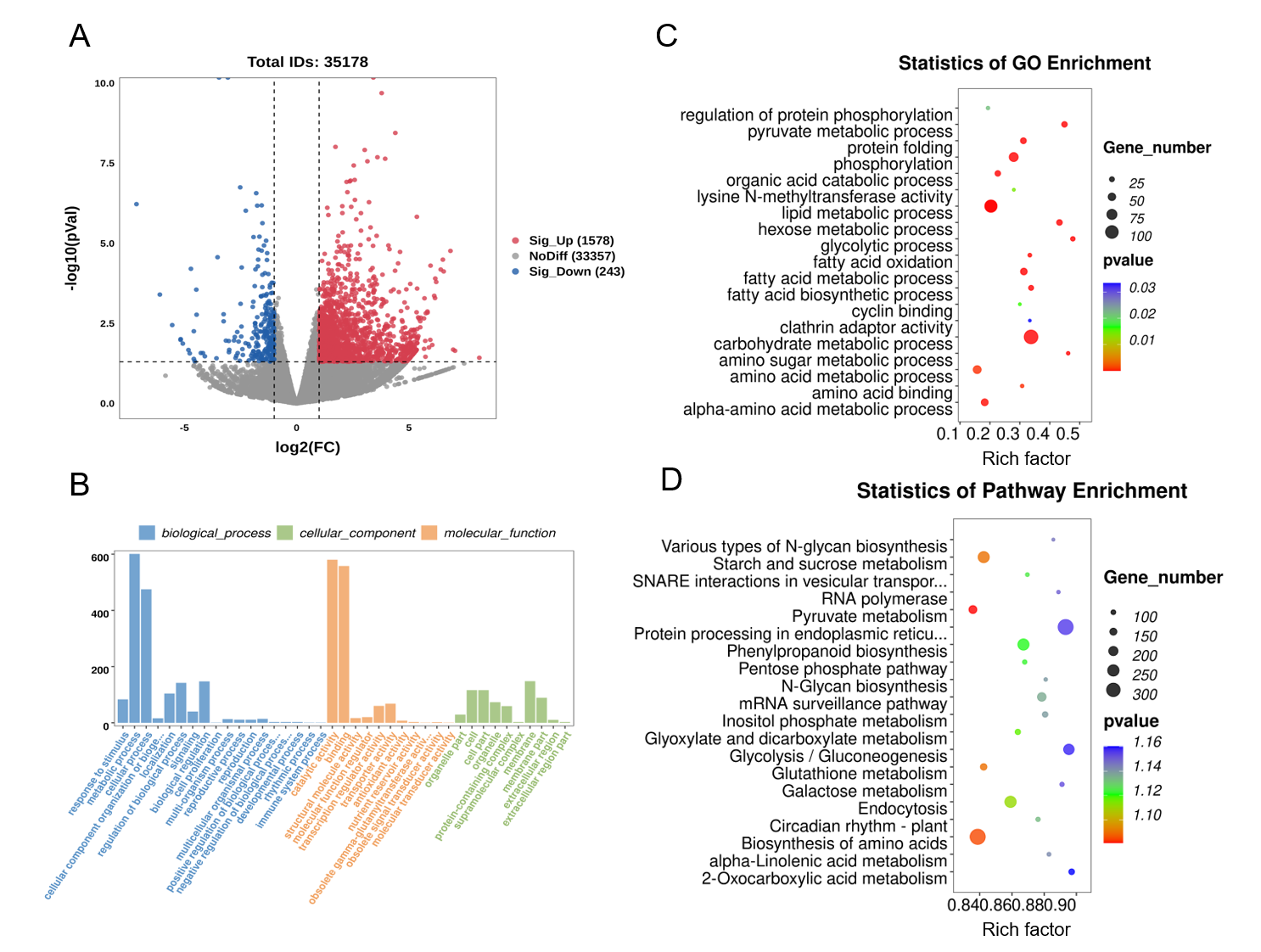

Supplement: Supplementary file 1 [file genes-17-00432-s001.zip › Figure S1. Comparative RNA-seq analysis between R19 and SN14..png]

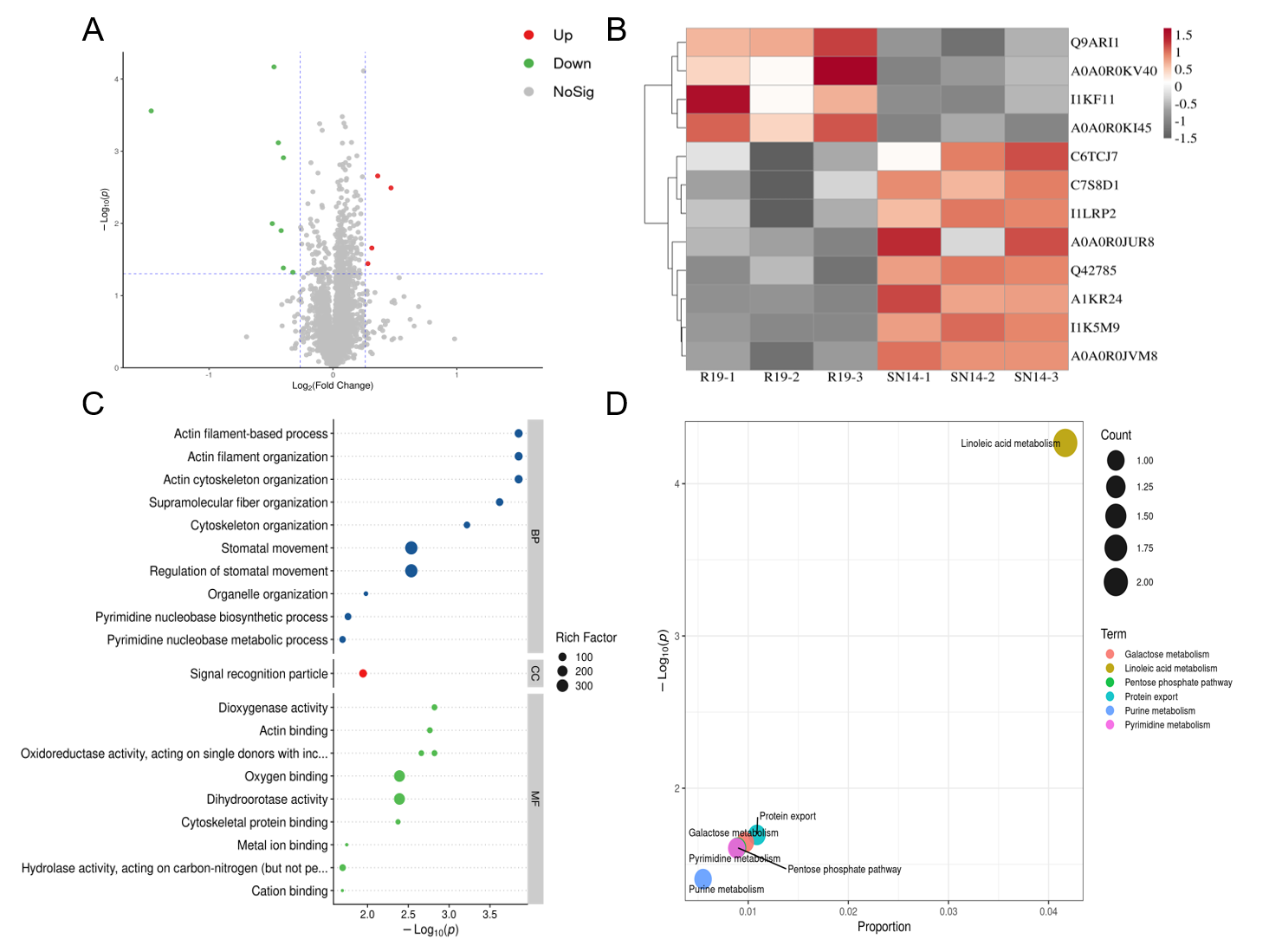

Supplement: Supplementary file 1 [file genes-17-00432-s001.zip › Figure S2. Comparative proteomic analysis between R19 and SN14..png]

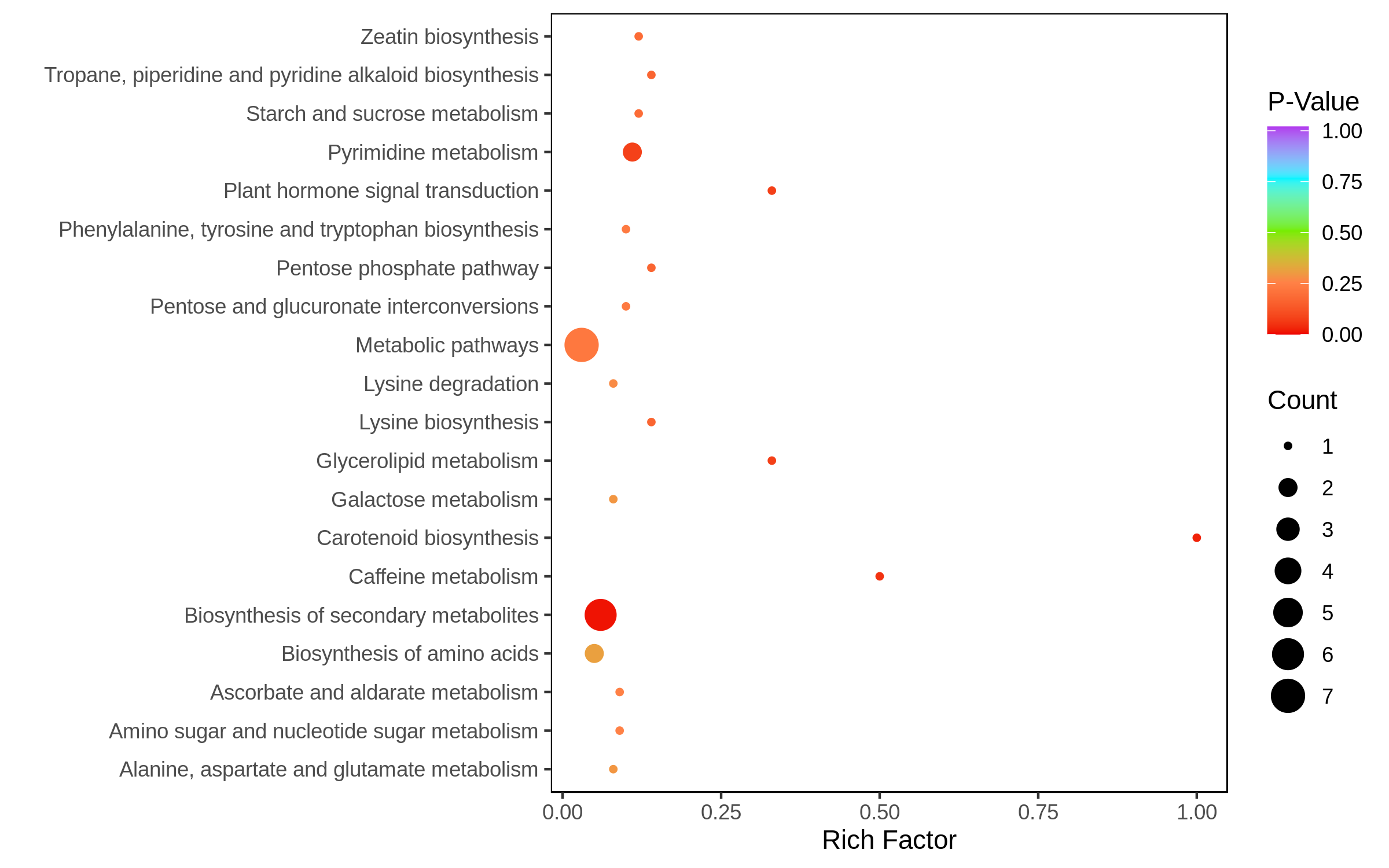

Supplement: Supplementary file 1 [file genes-17-00432-s001.zip › Figure S3. The KEGG enrichment analysis of DEMs between R19 and SN14..png]
